# Clinical Remarks on Surgical Cases Occurring at the Buffalo Hospital of the Sisters of Charity

**Published:** 1874-11

**Authors:** Julius F. Miner


ART. IV.—*Clinical Remarks upon Surgical Cases, occurring at the Buffalo Hospital of the Sisters of Charity.* By Prof. JULIUS F. MINER, M. D. Reported by W. W. MINER, M. D.

CASE IV.—*Granular Lids.*—J—— M——, aged 22, has suffered from sore eyes for several months. The cornea of the left eye has become quite inflamed, clouded, and vascular; the lids of both eyes are in a largely developed granular condition, and this is evident to you when they are everted. Vision is now considerably interfered with, and the prospects are that still more serious obstruction, or entire loss of vision will occur, and remain permanent, unless it is prevented.

Two thirds of the cases of eye disorders that you will have to treat, will be these of granular lids. The conjunctival lining of the everted lids will present these enlarged papilla, rough, inflamed and irritative to the delicate cornea, over which they are constantly being drawn with increased frequency of natural action. The cornea quickly becomes inflamed, clouded, and soon very markedly vascular; larger vessels at length coursing forwards on all sides towards the center of the cornea, and easily noticeable by the observer. Permanent opacity of the cornea, ulcerative perforation, and atrophy of the whole globe may result in these cases. Generally the result is much milder, but the condition is remarkably persistent when treatment is inadequate, or is altogether neglected.

The application of a crystal of sulphate of copper, is what you notice done in this case. Care is taken to bring the crystal in contact with every portion of the palpebral conjunctiva, as the operation is well nigh useless if any of the granulated membrane escapes reach. This application is to be repeated every day or two until a cure is effected. Patients will come to you blind, and in two weeks will see, under this treatment. Three months treatment is sometimes required. Nothing is better than sulphate of copper, and nothing else than it is needed. Practitioners are not confident enough in this; it is hard for my own students, even to learn that sulphate of copper is the only thing desirable in the treatment of granulations of the lid. Thorough application of it is the whole treatment and the cure of these cases.

If treatment is abandoned too early the patients will return with the trouble some months afterward. Considerable smarting is caused by the first application, but this diminishes with subsequent ones. Parents may think it harsh, but it is no more painful than many mild collyria, and these are generally ineffectual while with the copper you can have complete control of every case.

Case V.—*Syphilomania.*—Here is a man who had an attack of gonorrhœa, which came on in July and was soon shared in by his wife, both becoming rid of it in a reasonably short period. His present troubles are numerous, and he will best give you his own statement of them. He says that he suffers from pain across his bowels, passes water frequently, has spots or marks on his face, has numbness in head and hands, has a queer feeling which works down into his chest; his wife has the blood come into her face, neck swells up, dark around eyes; this he also has and his throat is somewhat sore.

He comes to me the very picture of dispair, is under the impression that he is awful sick, says: "See my face, all broke out, blood is all poisoned." I ask him what the matter is, and he answers: "Don't you see my face is all broke out with syphilis?" The gonorrhœa trouble which resulted from loose conduct was of a very mild type and though not confined to himself alone, it speedily ran its course and ended. With the urethritis however, the trouble has not all ended. Though no further bodily disorder is now apparent or to be expected in his case, still his mind is posse sed with the contrary idea.

Authors give little prominence to this condition, but these cases are frequently met with by those who are in a way to see much of this class of diseases. I tell him that he is insane on the subject, that unless he quits dwelling upon this matter he will be in the asylum for insane, that he is free from venereal disease and has no reason for thinking otherwise. One physician, of quite a number which he has consulted, writes me that he is too troublesome a fellow for him to get along with, and commends him unreservedly to my care. What is to be done with such patients? They are insane on the subject of their health and condition; they consult all kinds of doctors, and principally those quacks that encourage their absurdities and fears, and furnish them nostrums, specifics and placebos. They make up a large part of the subsistence of the quack profession. If they pay a good sum for advice, are gratified in their notions and furnished a panacea, they are easily manageable, whereas if they pay a moderate or small fee, are told the truth in a common manner, and sent away without medicine, they do not rest till they come to a man who manages them pretty shrewdly in some way or another. One of my colleagues suggests that this man is suffering under "compunctions of conscience," with which medical treatment will not greatly interfere. If he had syphilis certainly he would be little worse off than at present.

I could mention various cases where syphilis was experienced, or other venereal trouble, and a similar mental condition is manifested: when asked respecting their health they speak in the most despondent manner, saying they are nearly consumed, that they are dying and will soon be gone. This man is in just the suitable condition to be largely practiced upon, would no doubt pay anything he had in order to have the matter thoroughly attended to. This is generally the case; as long as their money lasts it will be spent in getting advice, consultation, medicine, humbug, or anything whatever that may seem in any way pertinent to their malady.

We cannot afford to dismiss such cases, neither ought we to leave them unconcernedly, a ready prey to quackery, imposture, unregulated or harmful medication. Salivation from "Sarsaparilla" is not by any means infrequent. You know pretty well what the course of these patients very generally is.

I have told this man what the real trouble with him is—have taken pains to bring him here for your examination—have obtained the opinion of my friend Professor Rochester, and others present. He will thus have the benefit of quite an amount of medical information and testimony. The proper course for him to pursue will be presented as impressively as circumstances will permit; how much effect upon him this may have, we can see much better hereafter.

Case VI.—*Erysipelas.* This man received a cut on his head some nine days since, and now presents an erysipelatous eruption on the ear, side of nose and head where the incised wound is situate. This eruption is always attended with heat, burning, smarting sensation in the parts affected, and is of more or less severity. Tincture of the perchloride of iron has been claimed as a specific, almost, in this affection. My opinion is that it will never do any harm and never much good. It may be taken in almost any quantities, but requires time to effect the system. Lead water, tincture of iodine, and various other external applications are, perhaps as good as water, seldom superior. Quinine and opium are the most important therapeutic remedies. I think medicines do not cut short its course. In a mild case like the present one, as the vast majority are, one would as willingly take the chances without medicine. Have seen serious cases, the eruption covering head, neck, a portion of the body; and there is then inflammatory fever of an active character. Opium is then beneficial and necessary. A specific for this we do not have; it has a regular course as does whooping cough, measles or other exanthemata, and generally terminates favorably.

Case VII.—*Trichiasis.* This woman has had an operation performed some time since for the removal of her eye-winkers. The rubbing of the hairs which fringe the lids upon the cornea may cause loss of vision. In injuries to the lids, this inverted condition not unfrequently results. The individual hairs may be pulled out to relieve the trouble, but this process needs to be repeated every few days, else the short hairs as they spring up will cause more irritation than those of natural length would. To radically remove the trouble, it is necessary that the hair bulbs be removed by excision, as has been very well done in this case; or an oval piece of palpebral integument removed; so that closing the wound will have a tendency to evert more or less the tarsal edge of the lid. Some degree of closure of the lids has been caused by the operation here performed, though it is less in degree than usually results from this method. The removal of an oval piece so as to produce proper eversion is more common, and where any extent of lid is inverted, the more desirable operation. A degree of inflammation and opacity which now troubles the patient, is due to granulation. Under the sulphate of copper treatment, she very speedily recovered from this and was dismissed from the hospital.

